# GCAP neuronal calcium sensor proteins mediate photoreceptor cell death in the rd3 mouse model of LCA12 congenital blindness by involving endoplasmic reticulum stress

**DOI:** 10.1038/s41419-020-2255-0

**Published:** 2020-01-24

**Authors:** Anna Plana-Bonamaisó, Santiago López-Begines, Jordi Andilla, María José Fidalgo, Pablo Loza-Alvarez, Josep María Estanyol, Pedro de la Villa, Ana Méndez

**Affiliations:** 1 Department of Physiological Sciences, University of Barcelona School of Medicine - Health Science Campus of Bellvitge, L´Hospitalet de Llobregat, 08907 Barcelona, Spain; 2 Institute of Neurosciences, University of Barcelona, Castelldefels, 08035 Barcelona, Spain; 3 Institut d’Investigació Biomèdica de Bellvitge ‐ IDIBELL, L´Hospitalet de Llobregat, Castelldefels, 08908 Barcelona, Spain; 4 ICFO-Institut de Ciències Fotoniques, The Barcelona Institute of Science and Technology, Castelldefels, 08860 Barcelona, Spain; 5 Centres Cientifics i Tecnològics (CCiTUB), University of Barcelona, Castelldefels, 08036 Barcelona, Spain; 6 Physiology Unit, Dept of Systems Biology, School of Medicine, University of Alcalá, Alcalá de Henares, 28805 Madrid, Spain; 7 Visual Neurophysiology Group-IRYCIS, Madrid, Spain

**Keywords:** Cell death in the nervous system, Retina, Neurodegenerative diseases

## Abstract

Loss-of-function mutations in the retinal degeneration 3 (*RD3*) gene cause inherited retinopathy with impaired rod and cone function and fast retinal degeneration in patients and in the natural strain of *rd3* mice. The underlying physiopathology mechanisms are not well understood. We previously proposed that guanylate cyclase-activating proteins (GCAPs) might be key Ca^2+^-sensors mediating the physiopathology of this disorder, based on the demonstrated toxicity of GCAP2 when blocked in its Ca^2+^-free form at photoreceptor inner segments. We here show that the retinal degeneration in *rd3* mice is substantially delayed by GCAPs ablation. While the number of retinal photoreceptor cells is halved in 6 weeks in *rd3* mice, it takes 8 months to halve in *rd3/rd3* GCAPs^−/−^ mice. Although this substantial morphological rescue does not correlate with recovery of visual function due to very diminished guanylate cyclase activity in *rd3* mice, it is very informative of the mechanisms underlying photoreceptor cell death. By showing that GCAP2 is mostly in its Ca^2+^-free-phosphorylated state in *rd3* mice, we infer that the [Ca^2+^]_i_ at rod inner segments is permanently low. GCAPs are therefore retained at the inner segment in their Ca^2+^-free, guanylate cyclase activator state. We show that in this conformational state GCAPs induce endoplasmic reticulum (ER) stress, mitochondrial swelling, and cell death. ER stress and mitochondrial swelling are early hallmarks of *rd3* retinas preceding photoreceptor cell death, that are substantially rescued by GCAPs ablation. By revealing the involvement of GCAPs-induced ER stress in the physiopathology of Leber’s congenital amaurosis 12 (LCA12), this work will aid to guide novel therapies to preserve retinal integrity in LCA12 patients to expand the window for gene therapy intervention to restore vision.

## Introduction

Synthesis of cyclic GMP (cGMP) is an essential process in photoreceptor cells of the retina, as cGMP is the signal-transducing molecule in the light response^1,2^. Mutations in a number of genes that impair or alter cGMP synthesis in rods and cones have been associated to different forms of blindness^3–12^. Loss-of-function mutations in the *RD3* gene (name from the natural strain of “retinal degeneration 3” mice, *rd3* locus mutated) cause Leber’s congenital amaurosis 12 (LCA12)^13,14^. LCA12 is characterized by rod and cone impaired function and severe vision loss from an early age, as well as rapid retinal degeneration.

The RD3 protein is required for the stability and ciliary trafficking of guanylate cyclases RetGC1 and RetGC2, responsible for cGMP synthesis^15^. In *rd3* mice the levels of RetGC1 and RetGC2 are dramatically decreased, and proteins are retained at the cell soma^15^. GCAPs (guanylate cyclase-activating proteins), that are proteins that confer Ca^2+^ sensitivity to RetGCs^16–20^ and depend on their binding to RetGCs for their stability and distribution to the outer segment, are also decreased in *rd3* mice^15,21,22^. As a consequence, there is reduced cGMP synthesis that results in closure of cyclic nucleotide-gated channels (CNG-channels) and presumed chronic hyperpolarization of *rd3* photoreceptors, concomitant to loss of visual function. This phenotype mimics that of LCA1 caused by null mutations in *GUCY2D* (RetGC1) in humans^23^, or by retinal guanylate cyclase deficiency in mice (RetGC1/RetGC2 double knockout mice^21^). However, while mice deficient in RetGC1/RetGC2 show a progressive retinal degeneration, in *rd3* mice the loss of photoreceptor cells progresses fast^24^.

RD3 was also reported to be a potent inhibitor of RetGC catalytic activity in vitro^25^, diminishing RetGC basal activity and competing with GCAP1 for RetGC binding. It was proposed that one role of RD3 would be to prevent RetGC activation while RetGCs traffic through the inner segment^25^.

Little is known about the molecular mechanisms that link the lack of RD3 with photoreceptor cell death in *rd3* mice. We previously proposed that the GCAP proteins could contribute to the physiopathology of retinal dystrophies characterized by rod/cone chronic hyperpolarization. This hypothesis was based on the fact that when a form of GCAP2 impaired to bind Ca^2+^ (with all functional EF-hands mutated, EF^−^GCAP2) was expressed in living photoreceptors, it was retained at the cell soma by phosphorylation and 14-3-3 binding, resulting in severe toxicity and fast retinal degeneration^26^. In *rd3* mice, GCAPs are retained at the cell soma in a presumed context of chronic low [Ca^2+^]_i_. In addition, GUCA1B (GCAP2) has been reported as a modifier gene of the *rd3* mouse phenotype^27^. We hypothesized that Ca^2+^-free GCAPs could be critically involved in the physiopathology of LCA12.

We here tested that hypothesis by breeding *rd3* mice to GCAPs^−/−^ mice. We show that the retinal degeneration of *rd3* mice was drastically delayed by GCAPs ablation. While in *rd3* mice the number of photoreceptors was halved in 6 weeks, in *rd3/rd3* GCAPs^−/−^ it was halved in 8 months. By assessing the extent of GCAP2 phosphorylation in *rd3* mice, we infer that the GCAP proteins are mostly in their Ca^2+^-free cyclase activator state in *rd3* cell somas. By expressing RD3.V5 as a transient transgene in the rods of *rd3* mice, we confirm that RD3 localizes mostly to the inner segment compartment of rods, which is consistent with the proposed role of RD3 as a RetGC inhibitor. We show prominent induction of endoplasmic reticulum (ER) stress and mitochondrial swelling in *rd3* mice, that are substantially prevented by GCAPs ablation. We conclude that GCAPs mediate the physiopathology of LCA12 by triggering ER stress, and discuss the putative mechanisms by which they might do so, ultimately causing mitochondrial swelling, energy failure, and cell death.

## Results

### Retinal degeneration due to RD3 deficiency is substantially rescued by GCAPs ablation

To test the hypothesis that the GCAP proteins contribute to the physiopathology of blindness associated to the lack of functional RD3, we bred *rd3* mice to GCAP1/GCAP2 double knockout mice (GCAPs^−/−^ mice), to assess whether the retinal degeneration was delayed.

**Fig. 1:**
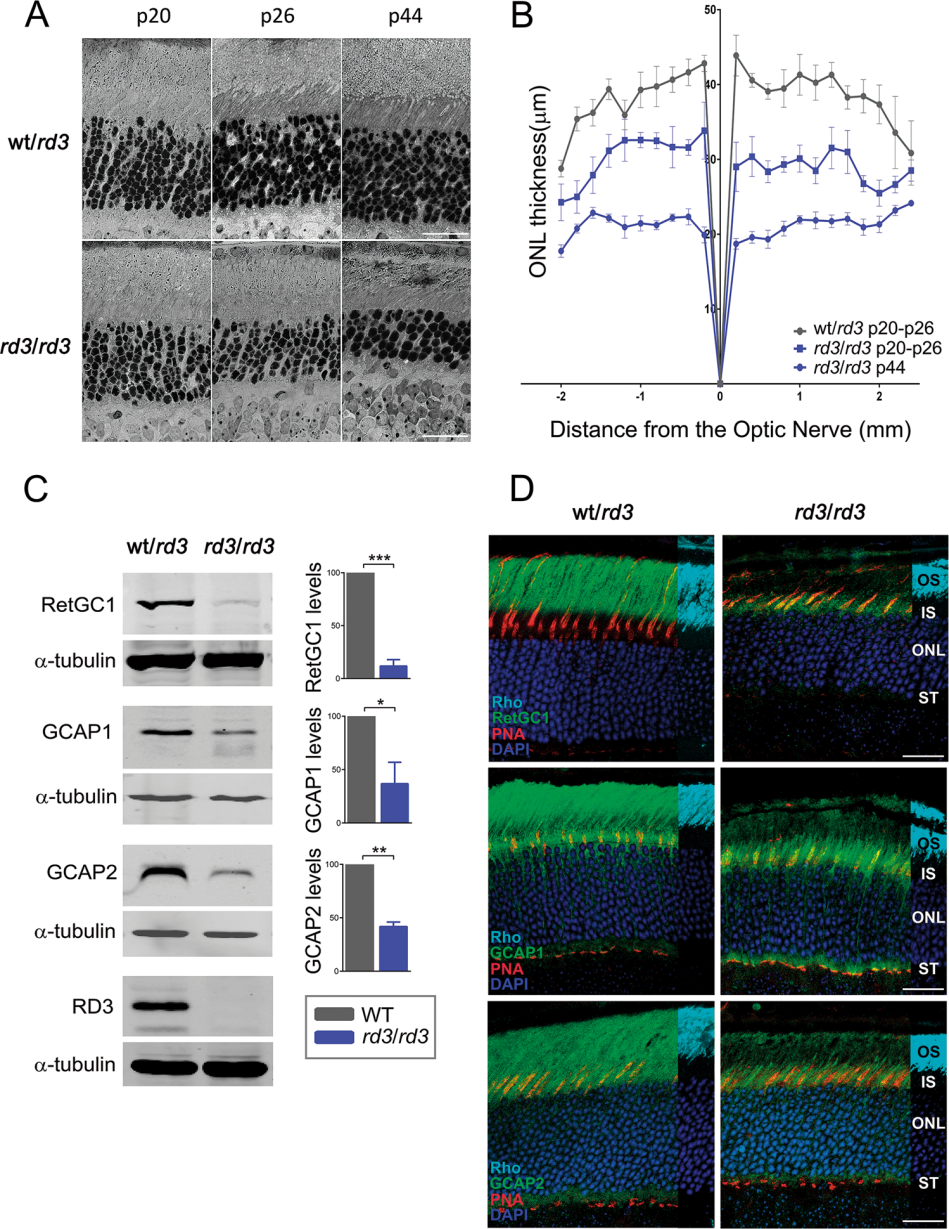
Time course and molecular alterations that characterize the retinal degeneration in *rd3* mice strain B6.Cg-Rd3rd3/Boc. **a** Retinal micrographs of wild type and *rd3/rd3* mice at p20, p26, and p44 show the progressive retinal degeneration in *rd3/rd3* mice, that halves the number of photoreceptor cells at p44. Scale bar 20 μm. **b** Retinal morphometric analysis of wt/*rd3* and *rd3/rd3* mice at the indicated ages, showing outer nuclear layer (ONL) length (μm) at 200 μm intervals from the optic nerve in the vertical meridian of the eye, superior retina to the right. Each trace represents the average measurements of four mice analyzed, with error bars indicating the standard error of the mean (SEM). (*n* = 2 biological replicas at p20, and *n* = 2 biological replicas at p26 for wt/*rd3* and *rd3/rd3*; *n* = 4 biological replicas at p44 for *rd3/rd3*). **c** Expression levels of RetGC1, GCAP1, and GCAP2 in *rd3/rd3* mice compared to wt/*rd3* mice at p30. Histograms indicate the percentage of expression of each protein in *rd3/rd3* mice versus wt/*rd3* mice, after normalization with α-tubulin. No RD3 protein is detected in *rd3/rd3* mice with an antibody generated against the RD3 C-terminus. For the determination of protein levels, three independent experiments were performed: RetGC1 (wt/*rd3* versus *rd3*; *P* < 0.0001***); GCAP1 (wt/*rd3* versus *rd3*; *P* < 0.032*); and GCAP2 (wt/rd3 versus *rd3*; *P* = 0.0017**). **d** Immunofluorescence localization of RetGC1, GCAP1, and GCAP2 in wt/*rd3* and *rd3/rd3* mice at p24. RetGC1, GCAP1, and GCAP2 signals are greatly reduced in *rd3/rd3* mice, and mostly restricted to the photoreceptor inner layer. Cones are labeled with peanut agglutinin (PNA) in red; the rod outer segment layer with anti-Rhodopsin mAb 1D4 (1 cm overlay in cyan), and nuclei are labeled with DAPI (blue). Scale bar 20 μm. OS, outer segment; IS, inner segment; ONL, outer nuclear layer; ST, synaptic terminals.

We first characterized the rate of retinal degeneration in the specific *rd3* strain used in this study (B6.Cg-Rd3rd3/Boc), hereinafter referred to as *rd3* mice, as rates of degeneration vary in different *rd3* strains^24^. As early as at postnatal day 20 (p20), *rd3* mice presented a thinner outer nuclear layer (ONL) than wild-type mice (10 rows versus 12 rows of nuclei, Fig. 1a). An statistical analysis of ONL thickness performed on morphometric measurements from wt/*rd3* and *rd3/rd3* littermate mouse retinas at p20, p26, and p44 revealed that in *rd3* mice the number of photoreceptor cells was halved in ~6 weeks (Fig. 1a, b). This is consistent with the reported rate of degeneration of *rd3* pigmented mouse strains^24^.

We confirmed that RetGC1 expression was severely reduced in *rd3* mice^15^. RetGC1 levels were reduced to ~12% of the expression in control mice at p20, Fig. 1c. The expression levels of GCAP1 and GCAP2 were reduced to 37 and 42% of the levels in control mice, Fig. 1c. A novel observation was that RetGC1 was detected by immunofluorescence analysis on retinal sections of *rd3* mice at the inner segment layer and perinuclear region of photoreceptors, showing a characteristic punctated staining, Fig. 1d. GCAP1 and GCAP2 were largely retained to proximal photoreceptor compartments in *rd3* mice, as previously described^15^, Fig. 1d. Specificity of the antibodies to RetGC1 and RD3 used in Fig. 1c, d is shown in Supplementary Fig. 1.

**Fig. 2:**
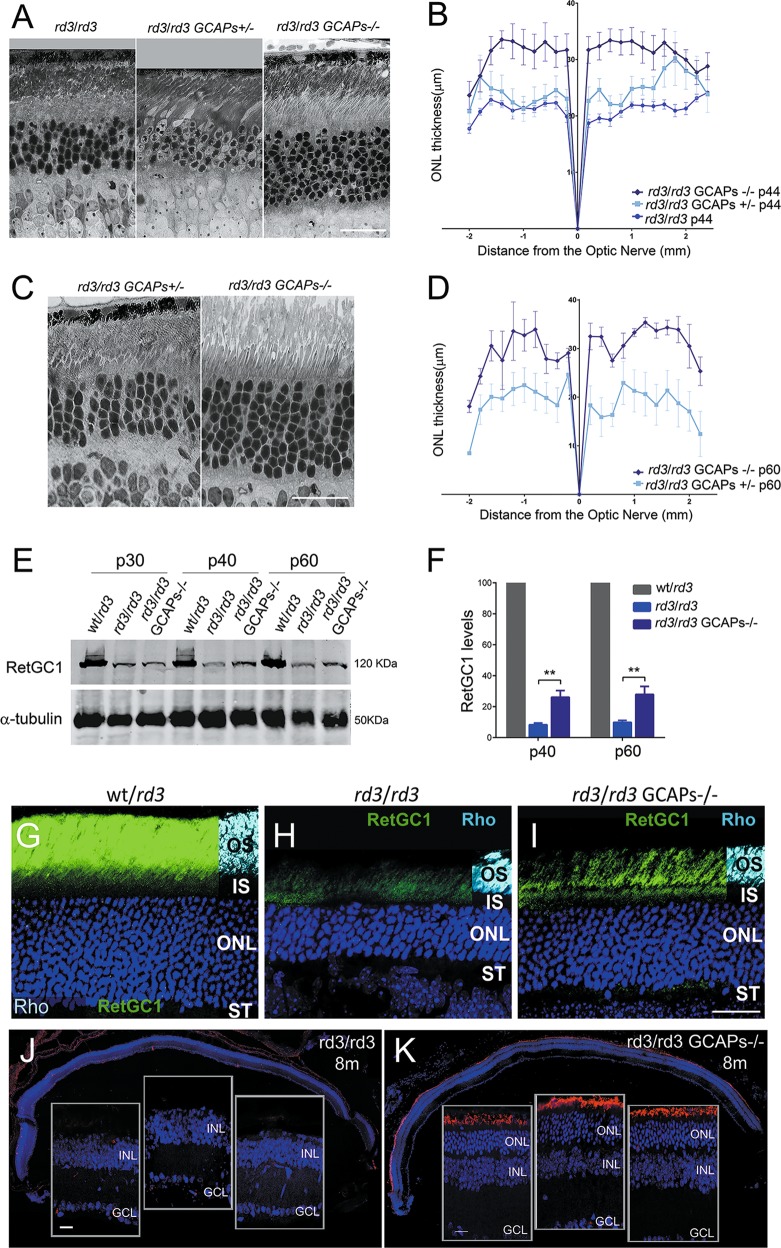
Retinal degeneration in *rd3* mice is substantially prevented by GCAPs ablation. **a** Retinal morphology of *rd3/rd3*; *rd3/rd3* GCAPs^+/−^ and *rd3/rd3* GCAPs^−/−^ mice at p44. Photoreceptor cell loss is substantially prevented in the GCAPs^−/−^ background. Scale bar 20 μm. **b** Retinal morphometry analysis of the indicated genotypes at p44 showing ONL length (μm) along the vertical meridian of the eye. Each trace shows the average from measurements taken from four mice, with error bars showing the standard error of the mean (SEM). GCAPs removal results in preservation of 25% more photoreceptors at p44. **c** Morphology of the retina in *rd3/rd3* GCAPs^+/−^ and *rd3/rd3* GCAPs^−/−^ at p60. The protective effect of GCAPs ablation persists at this age. Scale bar 20 μm. **d** Statistical analysis of ONL length in *rd3/rd3* GCAPs^+/−^ and *rd3/rd3* GCAPs^−/−^ retinas at p60. Results are mean ± SEM of four biological replicas. **e** Level of expression of RetGC1 in *rd3/rd3* GCAPs^+/−^ and *rd3/rd3* GCAPs^−/−^ retinas at p30, p40, and p60. RetGC1 expression levels correlate with the fraction of photoreceptors preserved. **f** Statistical analysis of RetGC1 expression levels, mean ± SEM of three biological replicas per genotype. RetGC1 levels are not altered by the presence or absence of GCAPs at p30, consistent with *rd3/rd3* and *rd3/rd3* GCAPs^−/−^ mice having a similar ONL thickness at this age. RetGC1 levels are significantly increased in the GCAPs^−/−^ background at p40 and p60, reflecting the extent to which photoreceptor cells are preserved at these ages. Unpaired *t*-test (*rd3/rd*3 versus *rd3/rd3* GCAPs^−/−^ at p40, *P* = 0,0023**); (*rd3/rd3* versus *rd3/rd3* GCAPs^−/−^ at p60, *P* = 0,0043**). **g**–**i** In the absence of GCAPs, more RetGC1 distributes to the rod outer segment (ROS) layer in *rd3* mice **i**, than in the presence of GCAPs **h**. Scale bar 20 μm. OS, outer segment; IS, inner segment; ONL, outer nuclear layer; ST, synaptic terminals. **j**, **k** The protective effect of GCAPs ablation persists at 8 months. A representative retinal section of *rd3/rd3* mice at 8 months, with nuclei stained with DAPI (blue), shows a complete loss of the photoreceptor cell layer **j**. A retinal section of *rd3/rd3* GCAPs^−/−^ mice at 8 months shows an outer nuclear layer with five rows of nuclei that preserve their outer segments (note the ROS layer stained with anti-rhodopsin antibody). Retinal sections are representative of three mice per genotype. Scale bar 20 μm.

To assess whether ablation of GCAPs affected the time course of retinal degeneration in the *rd3* mice, we bred *rd3* mice to GCAPs^−/−^ mice. To minimize strain variation effects, the breeding was established between *rd3/rd3* GCAPs^+/−^ mice, and homozygous *rd3/rd3* mice that were either GCAPs^+/−^ or GCAPs^−/−^ littermates were compared, Fig. 2. The ONL width of *rd3/rd3* mice at p44 from Fig. 1 is plotted as a reference. Compared to *rd3/rd3* mice at p44 (ONL thickness of 20 μm, 5 nuclei), *rd3/rd3* GCAPs^+/−^ showed a very minor improvement at this age (22–24 μm, 5–6 nuclei, *n* = 4); while *rd3/rd3* GCAPs^−/−^ mice showed a striking improvement (30–32 μm, 7–8 nuclei, *n* = 4), Fig. 2a, b. This substantial rescue effect of GCAPs ablation was preserved at p60 (Fig. 2c, d), when four biological replicas per genotype were analyzed. In the absence of GCAPs, the *rd3* mice preserved 75% of their photoreceptors at p60; while in the presence of GCAPs they preserved <50%.

This substantial preservation of photoreceptors in the absence of GCAPs correlated with an increase in RetGC1 levels in *rd3/rd3* GCAPs^−/−^ retinas (Fig. 2e, f). Quite surprisingly, RetGC1 in *rd3/rd3* GCAPs^−/−^ mice was distributed to rod outer segments (ROSs) to a higher extent than in *rd3/rd3* mice (Fig. 2g, i).

At 8 months of age, *rd3/rd3* mice did not retain any photoreceptor cells, while *rd3/rd3* GCAPs^−/−^ mice preserved five rows of photoreceptors with visible outer segments, Fig. 2j, k.

We conclude that GCAPs ablation markedly slows down photoreceptor cell death in the *rd3* mice, implying that GCAP proteins are involved in the physiopathology of LCA12.

### Morphological rescue of rd3 retinas by GCAPs ablation does not correlate with an amelioration of visual function

To test whether the substantial morphological rescue of *rd3* mice that resulted from GCAPs ablation correlated with an amelioration of visual function, light-elicited electroretinogram (ERG) responses were recorded from wt, *rd3/rd3*, GCAPs^−/−^, and *rd3/rd3* GCAPs^−/−^ mice at p40.

**Fig. 3:**
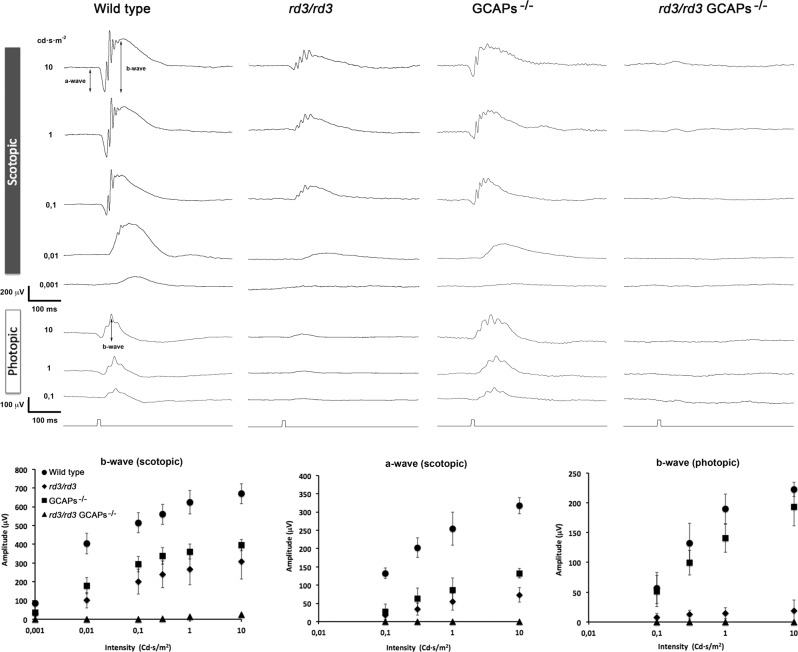
Light responses are drastically reduced in *rd3/rd3* GCAPs^−/−^ mice. Electroretinographic recordings from 40-d-old wt, *rd3/rd3*, GCAPs^−/−^, and *rd3/rd3* GCAPs^−/−^ mice in response to light stimuli of increasing intensity at scotopic or photopic conditions. Representative recordings are shown from one mouse of each genotype, evoked by light pulses of increasing intensity (indicated in cd/s/m^2^). Scotopic a-wave and b-wave amplitudes (mean ± SD) and photopic b-wave amplitudes are plotted at different light intensities from a total of four wt mice, seven *rd3/rd3* mice, five GCAPs^−/−^, and five *rd3/rd3* GCAPs^−/−^ mice. Both GCAPs^−/−^ and *rd3/rd3* mice showed substantially reduced responses when compared to wild-type responses. GCAPs ablation in *rd3/rd3* mice resulted in nearly abolished responses at the scotopic or photopic range.

*Rd3* mice showed substantially reduced scotopic and photopic responses (Fig. 3), as expected based on the drastically reduced RetGC levels in these mice, as previously reported^28^. Despite this substantially reduced sensitivity to light, *rd3* mice elicited diminished but reliable responses to flashes in the scotopic and photopic range (Fig. 3), indicating that at p40 they retained some rod and cone visual function.

GCAPs^−/−^ mice presented reduced visual responses in the scotopic and photopic range due to the lack of GCAPs stimulation of guanylate cyclase activity, but they largely retained visual function (Fig. 3).

Strikingly, *rd3/rd3* GCAPs^−/−^ mice at p40 yielded barely noticeable responses to flashes at either the scotopic or photopic range, despite maintaining 25% more photoreceptor cells than *rd3/rd3* mice and higher levels of RetGC at their retinas. A possible explanation for these results is that *rd3/rd3* GCAPs^−/−^ responses reflect the lack of stimulation of RetGC activity by the GCAP proteins; whereas *rd3/rd3* mice retain GCAP stimulation of the remaining RetGC proteins.

Taken together our results show that GCAPs ablation in the *rd3* mice substantially prevented retinal degeneration but at the cost of impairing rather than ameliorating visual function.

### Subcellular localization of RD3 in retinal sections

RD3 was described as a potent inhibitor of RetGC activity in vitro, and was predicted to localize to the inner segment layer where it could silence the cyclase during its transport to the cilium^25^. However, RD3 was immunolocalized to the outer segment layer of the retina in wt mice by specific antibodies against the COOH-terminus of the protein^15^.

To determine RD3 subcellular localization by avoiding the use of anti-RD3 antibodies, we generated transient transgenic mice that expressed RD3 fused to a short tag (RD3.V5) in rod photoreceptors. To express RD3.V5 in rods, we made use of in vivo DNA electroporation after subretinal injection in newborn *rd3/rd3* mice with the construct pRho-RD3.V5-dsRed. RD3 was then immunolocalized at p20 using well-established anti-V5 antibodies (Methods section).

**Fig. 4:**
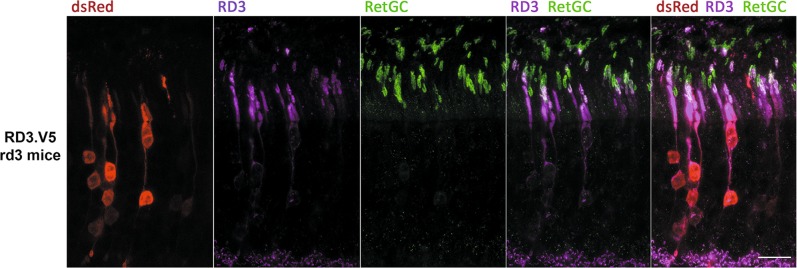
Subcellular localization of RD3 protein in murine retinas. Immunolocalization of RD3.V5 protein in retinal sections from *rd3/rd3* mice electroporated with pRho-mRD3.V5-dsRed. RD3.V5 distributes between the inner and outer segment compartments of transfected photoreceptor cells, with a stronger signal at inner segments. Note that the expression of RD3.V5 in transfected cells restores RetGC1 expression levels and its transport to the outer segment. The red channel shows the dsRed signal from pRho-dsRed, a plasmid coelectroporated with pRho-mRD3.V5-dsRed to serve as a reporter in the identification of the eye injected area (Methods section). Anti-V5 antibody staining of the synaptic ribbons is observed in transfected and untransfected cells, and is an unspecific signal. Scale bar 20 μm.

RD3.V5 showed the mosaic pattern of expression that characterizes in vivo DNA electroporation transgenesis, in which only transfected cells express the transgene. The expression of RD3.V5 in *rd3/rd3*-transfected rods restored RetGC1 expression and its localization to the outer segment, which indicated that the RD3.V5 protein was active (Fig. 4). RD3.V5 localized to the inner and outer segments of transfected cells, giving a much stronger signal at the inner segments. Particularly, there appears to be a membrane domain, probably at the connecting cilium, where the RetGC1 and RD3.V5 signals strongly colocalize (see the white signal from the merged green and magenta channels with “staple” shape, Fig. 4).

Therefore our results are in line with the results obtained by Dizhoor et al.^29^ that localize RD3 mainly at the inner segment, although we observe some signal at the outer segment as well. The mosaic expression excludes that this signal is unspecific, as electroporated cells are surrounded by negative control cells. Anti-V5 antibodies unspecifically stain synaptic ribbons. This signal is clearly unspecific because it is detected in transfected and untransfected cells (Fig. 4).

### GCAP2 in *rd3* mice is mostly in its phosphorylated Ca^2+^-free form, a target for 14-3-3 binding

We have previously reported that an accumulation of Ca^2+^-free GCAP2 at the inner segment is highly deleterious for rod cells^26^. In *rd3* photoreceptor cells, GCAP2 is retained at the inner segment (Fig. 1d). To test whether GCAP2 is in its Ca^2+^-free “deleterious” conformation in *rd3* mice, we analyzed GCAP2 levels of phosphorylation.

In vitro and in vivo studies have shown that GCAP2 is phosphorylated at Ser201 preferentially in its Ca^2+^-free form^26,30^. In wt mice, ~50% of GCAP2 is phosphorylated when mice are reared under standard cyclic light, independently of their sacrifice in the dark or light period^26^. However, a GCAP2 mutant locked in its Ca^2+^-free form (EF^−^GCAP2) transgenically expressed in rods showed a much higher extent of GCAP2 phosphorylation. This indicates that the extent of GCAP2 phosphorylation in vivo reflects the fraction of GCAP2 molecules in the Ca^2+^-free conformation^26^.

**Fig. 5:**
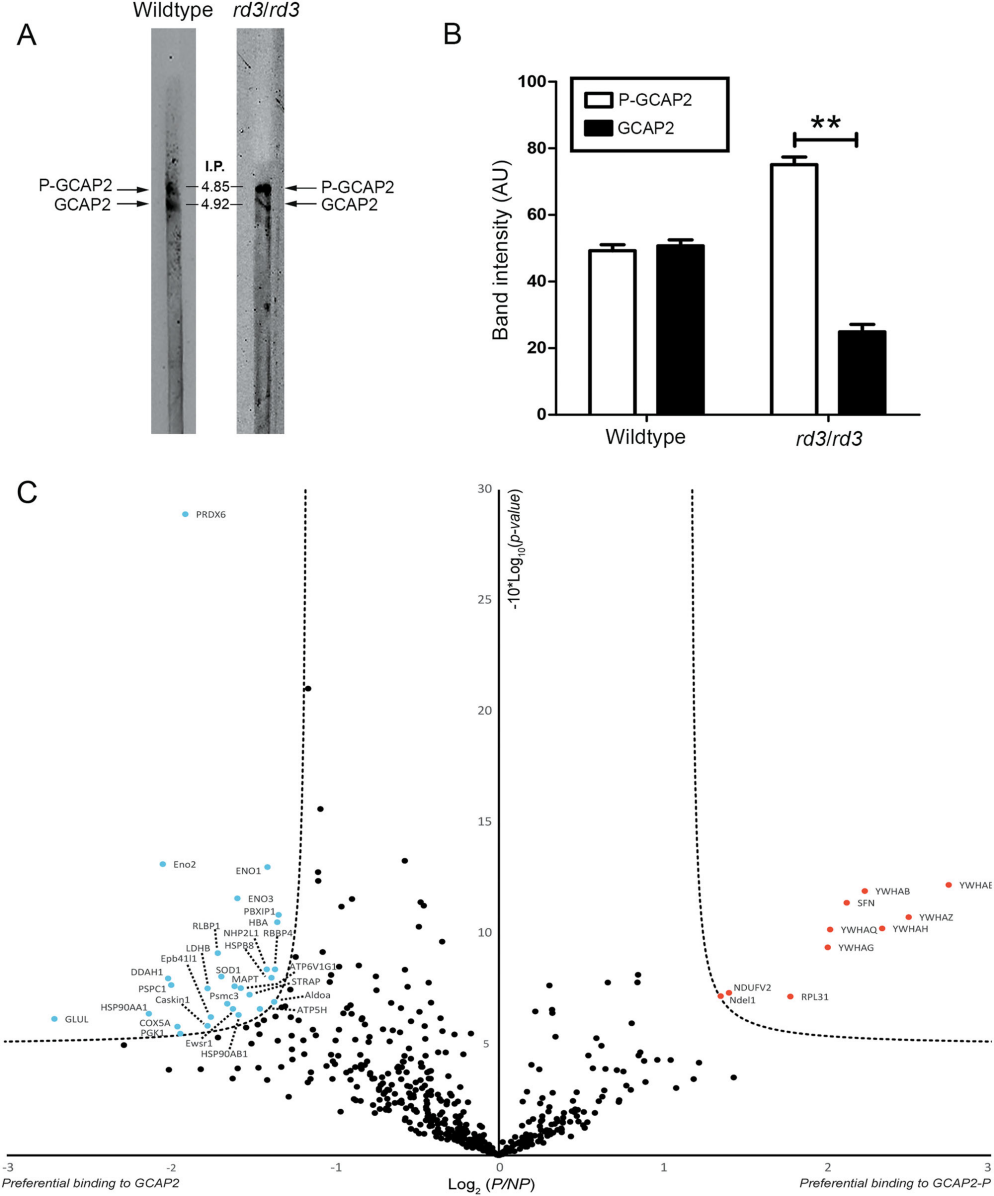
GCAP2 in *rd3/rd3* retinas is largely in its Ca^2+^-free-phosphorylated state, which is a target for 14-3-3 binding. **a** Isoelectric focusing (IEF) separation of retinal homogenates from wt and *rd3/rd3* mice at p30 in linear pH 3–10 gradient gel strips. Following IEF, proteins were transferred to a nitrocellulose membrane and immunoblotted with anti-GCAP2 pAb. **b** The GCAP2-P to GCAP2 ratio was determined by densitometric analysis (mean ± SEM) of three independent isoelectrofocusing experiments. Unpaired *t*-test (GCAP2 versus GCAP2-P in wt mice, *P* = 0.72) and (GCAP2 versus GCAP2-P in *rd3* mice, *P* = 0.0082**). **c** Label-free quantitative proteomic analysis to identify putative protein interactions of Ca^2+^-free GCAP2-P and GCAP2. Pull-down assays were performed on bovine retinal homogenates with purified myristoylated GCAP2 that was phosphorylated in vitro or a MOCK-phosphorylation control, under EGTA conditions. The volcano plot represents the statistical analysis of three independent pull-down assays. Log2 (*P*/*NP*) refers to Log2 (Mean NSAF_GCAP2-P_/Mean NSAF_GCAP2_), with NSAF being the normalized spectral abundance factor, see Methods section. The already reported 14-3-3 phosphobinding proteins (different isoforms) were clearly revealed as Ca^2+^-free GCAP2-P interactors, validating the assay. Other putative interactors identified with statistical significance are marked in red (GCAP2-P) and blue (GCAP2). The whole list of identified proteins is provided in Supplementary Table 1.

The extent of GCAP2 phosphorylation in *rd3/rd3* versus wt mice was analyzed by resolving retinal homogenates at p21 in isoelectrofocusing gel strips that covered a linear pH gradient of 3–10 (Fig. 5a). Threefold more *rd3/rd3* protein sample (100 μg) than wt sample (25 μg) was loaded, in order to equilibrate the GCAP2 signal. Unphosphorylated GCAP2 presented an isoelectric point (IP) of 4.92, whereas phosphorylated GCAP2 an IP of 4.85. In wt mice, GCAP2 and GCAP2-P were present at a 1:1 ratio as originally reported. In contrast, GCAP2 and GCAP2-P were observed at a 1:3 ratio in *rd3* mice (Fig. 5b). This ratio of GCAP2 to GCAP2-P was very similar to the ratio reported for EF^−^GCAP2 mice^26^, and indicates that GCAP2 in *rd3* mice is mostly in its phosphorylated Ca^2+^-free state.

To gain insight into the mechanism by which Ca^2+^-free GCAP2 could contribute to the physiopathology of *rd3* mice, we searched for molecular targets of Ca^2+^-free GCAP2 in the retina. We performed pull-down assays with purified myristoylated recombinant bGCAP2 that was in vitro phosphorylated—or with its mock control—on Triton-X100 solubilized bovine retinas, under EGTA conditions (Methods section). Experiments were performed in triplicate. Bound proteins were identified by liquid chromatography and mass spectrometry (LC-MS/MS) and subjected to label-free quantitative statistical analysis. For each identified protein (776 total proteins, Supplementary Table 1), the equation detailed in Methods section was used to determine its normalized spectral abundance factor (NSAF) in the GCAP2-P and the GCAP2 sample. The scatter plot in Fig. 5c presents the Log_2_ (Mean NSAF_GCAP2-P_/Mean NSAF_GCAP2_) on the *x*-axis, versus the −10Log(*P* value) in the *y*-axis, considering threshold values of 1,35 and 5 for the *x*- and *y*-axis, respectively. Proteins that showed an statistically significant preference for GCAP2-P are shown as red dots, while those with preference for unphosphorylated GCAP2 as blue dots (Fig. 5c).

The 14-3-3 proteins, known to interact with GCAP2-P^26^, were identified with robust statistics as GCAP2-P binding proteins; as were proteins NDUFS5 (NADH dehydrogenase(ubiquinone) iron-sulfur protein 5), Ndel1 (nuclear distribution protein nude-like1), and RPL31 (60S ribosomal protein L31). On the other side, among the proteins with preference for unphosphorylated Ca^2+^-free GCAP2, were HSP90(α,β) and HspB8, chaperones that may be required to stabilize this unstable form of the protein, and superoxide dismutase 1 and peroxiredoxin-6, involved in maintaining the redox state of the cell.

RetGC1 was only identified in two out of three experiments, with ≤6 spectral counts, and showed a slight preference for the unphosphorylated form of GCAP2 (Supplementary Table 1). However, it is well established that the RetGC–GCAPs interaction is very sensitive to detergents, and virtually impossible to detect in pull-down assays.

### ER stress and mitochondrial swelling are prominent early signs of retinal degeneration in the *rd3* mice that are substantially rescued by GCAPs ablation

**Fig. 6:**
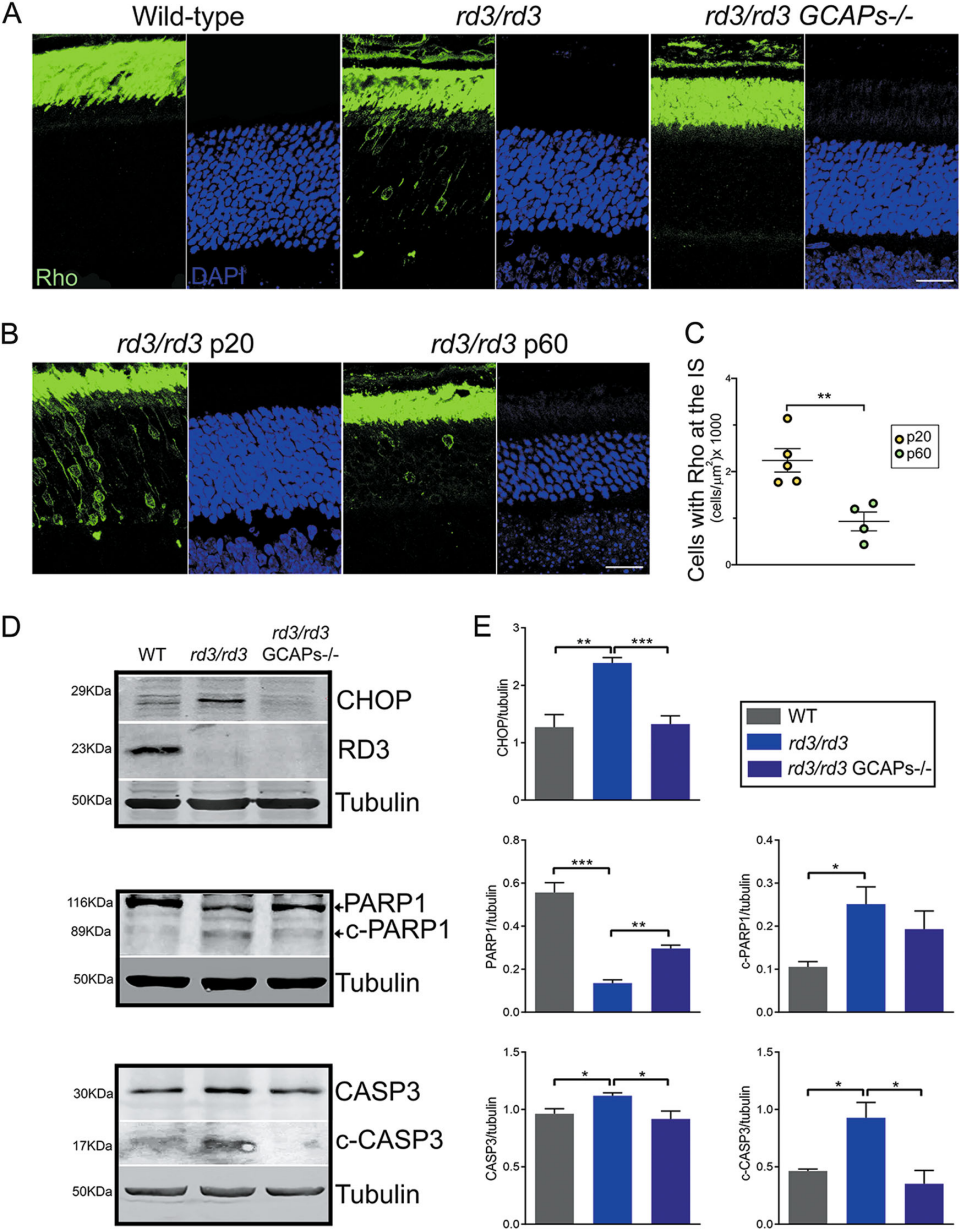
Rhodopsin mislocalization, ER stress, and apoptosis are early signs of retinal degeneration in *rd3* mice that are palliated by GCAPs ablation. **a** Retinal sections from wt, *rd3/rd3*, and *rd3/rd3* GCAPs^−/−^ mice at p20 stained with anti-rhodopsin antibody (green) and DAPI (blue). Rhodopsin mislocalization at the perinuclear region and proximal compartments is observed in a number of photoreceptor cells at any given frame of the outer retina in *rd3/rd3* mice at p20, but not in wt or *rd3/rd3* GCAPs^−/−^ mice this age. Scale bar 20 μm. **b**, **c** Representative images and quantification of cells that present rhodopsin mislocalization at p20 versus p60 in *rd3* mice, expressed per unit area (*n* = 5 *rd3/rd3* mice at p20; *n* = 4 *rd3/rd3* mice at p60]. Unpaired *t*-test p20 versus p60, *P* = 0.0058**). **d** Levels of CHOP; full length and p17 kDa fragment of cleaved caspase 3; and full length and 89 kDa fragment of cleaved PARP1 proteins in retinal extracts from wt, *rd3/rd3*, and *rd3/rd3* GCAPs^−/−^ at p20. Note that the full-length casp3 and c-casp3 signals were obtained from different exposure conditions of the same membrane, given that c-casp3 represents a small percentage of full-length casp3. No RD3 protein was observed in *rd3/rd3* or *rd3/rd3* GCAPs^−/−^ extracts. **e** Six independent experiments were performed to determine CHOP expression levels (wt versus *rd3/rd3*, *P* = 0.0012**; and *rd3/rd3* versus *rd3/rd3* GCAPs^−/−^, *P* = 0.0001***). Three independent experiments were performed to determine PARP1 and c-PARP1 levels (PARP1: wt versus *rd3*, *P* = 0.001***; and *rd3/rd3* versus *rd3/rd3* GCAPs^−/−^, *P* = 0.0018**); (c-PARP1: wt versus *rd3*, *P* = 0.026*; and *rd3/rd3* versus *rd3/rd3* GCAPs^−/−^, *P* = 0.37 NS). Three independent experiments were performed to determine casp3 and c-casp3 levels (casp3: wt versus *rd3*, *P* = 0.035*; and *rd3/rd3* versus *rd3/rd3* GCAPs^−/−^, *P* = 0.05*); (c-casp3: wt versus *rd3*, *P* = 0.026*; and *rd3/rd3* versus *rd3/rd3* GCAPs^−/−^, *P* = 0.031*).

One early alteration that we observed in *rd3* retinas at p20 was rhodopsin mislocalization. Although rhodopsin transport is not affected by the lack of RD3 and rhodopsin antibodies largely stain the outer segment layer in *rd3* mice (e.g., Fig. 1), a number of photoreceptor cells showed rhodopsin perinuclear staining in any taken frame of the *rd3* outer retina at p20 (Fig. 6a). This was not observed in age-matching wt or *rd3/rd3* GCAPs^−/−^ mice (Fig. 6a). The number of cells per unit area that showed mislocalized rhodopsin in *rd3* mice was highest at p20 and diminished with age (Fig. 6b, c).

Because this signal could be reflecting rhodopsin retention at the ER due to ER dysfunction, we assessed ER stress by comparing the levels of ER stress marker C/EBP homologous protein (CHOP) in wt, *rd3/rd3* and *rd3/rd3* GCAPs^−/−^ mice. CHOP is a transcription factor that links severe ER impairment with the induction of apoptosis^31^. We observed a clear induction of CHOP expression in *rd3* mice at p20 (Fig. 6d, e), that was substantially prevented by GCAPs ablation (Fig. 6d, e).

Apoptotic cell death was assessed by evaluating caspase 3 activation (detection of the p17 large fragment from caspase 3 cleavage) and the cleavage of poly (ADP-ribose) polymerase PARP, a main target of caspases. Figure 6d, e shows activated p17 fragment of caspase 3 at a much higher level in *rd3* mice than in wt or *rd3/rd3* GCAPs^−/−^ mice, with statistical significance (experiment performed in triplicate). The extent of PARP1 cleavage in *rd3* mice versus wt and *rd3/rd3* GCAPs^−/−^ mice confirms the induction of apoptotic cell death in *rd3* mice at p20, that is partially rescued by GCAPs ablation.

**Fig. 7:**
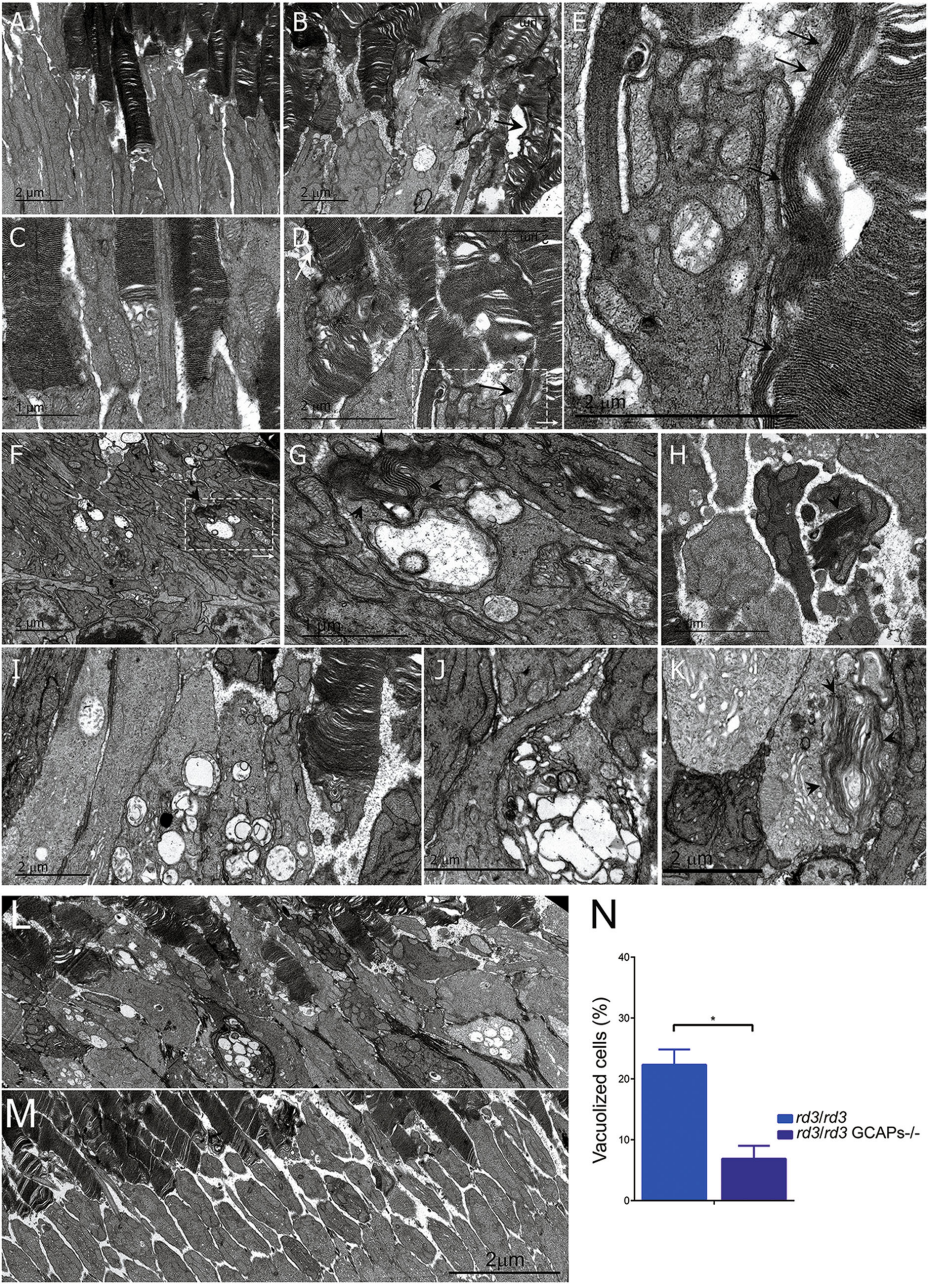
Mitochondrial swelling is an early sign of retinal degeneration in *rd3/rd3* mice that is substantially prevented by GCAPs ablation. **a**–**e** Comparison of *rd3/rd3* retinas at p20 (**b**, **d**) versus *rd3*/wt littermate controls (**a**, **c**). Retinas from *rd3/rd3* mice present distinctive features like emerging vertical outer segment membrane discs (black arrows in **b** and **d**, and in the enlarged area in **e**); as well as swelling mitochondria (arrowheads, **b** and **e**). **f**, **g**, **i**, **j** Mitochondrial swelling is a landmark ultrastructural alteration in *rd3/rd3* mice at early stages of retinal degeneration, as observed dramatically at a high number of photoreceptor cells at p26. **h**, **k** Other striking alterations are the internalization at the inner segment of what appears to be stacks of outer segment disc membranes (black arrowheads). **l**, **m**, **n** Mitochondrial swelling is substantially prevented by GCAPs ablation in the *rd3* mice, as observed by comparing a representative electron micrograph of retinal sections from *rd3/rd3* **l** and *rd3/rd3* GCAPs−/− mice **m** at p26. Images are the result of fusing four transmission electron microscopy fields obtained at 10,000× magnification. The number of photoreceptor cells that present mitochondrial swelling is substantially reduced in *rd3/rd3* GCAPs^−/−^ mice versus *rd3/rd3* mice. **n** Four independent regions were analyzed per retina; and retinas were obtained from four mice of each genotype. Histograms represent the percentage of cells with presence of vacuoles, from the total number of cells. Unpaired *t*-test (*rd3/rd3* versus *rd3/rd3* GCAPs^−/−^, *P* = 0.019*).

Actually, one of the most prominent alterations in *rd3* mice at p20–p26 revealed by ultrastructural analysis was mitochondrial swelling, indicative of apoptosis. Figure 7 shows representative electron micrographs of *rd3/rd3* mice at p20–p26 (Fig. 7b, d), opposed to *rd3*/wt heterozygous littermate controls (Fig. 7a, c). Retinas from *rd3/rd3* mice showed distinctive features like emerging vertical outer segment membrane discs (black arrows in Fig. 7b, d, and enlarged area in Fig. 7e); as well as disc structures that have been internalized at the inner segment (black arrowheads in Fig. 7f, g, h, k). The most prominent morphological alteration in *rd3/rd3* mice was mitochondrial swelling at the inner segment, noticeable at p20 at a fraction of cells (Fig. 7f, magnified at Fig. 7g), and at p26 at the majority of cells (Fig. 7i, j). This mitochondrial swelling preceded cell death.

Interestingly, the striking mitochondrial swelling in *rd3* photoreceptor cells (Fig. 7l) was greatly diminished in *rd3/rd3* GCAPs^−/−^ mice (Fig. 7m) when four animals of each genotype were compared (Fig. 7n).

Taken together, our results show that GCAPs induce the ER stress response in *rd3* mice, inducing CHOP expression and triggering apoptosis at a very early stage of the disease, and that GCAPs ablation substantially suppresses this induction and prevents photoreceptor cell death.

## Discussion

We here show that the GCAP proteins play a central role in the physiopathology of blinding diseases associated to loss of functional RD3 (LCA12), by showing that GCAPs ablation substantially delays retinal degeneration in the *rd3* mouse model. While preservation of the retinal morphology is not accompanied by restoration of visual function, it is very revealing of the molecular mechanisms that link the lack of functional RD3 with photoreceptor cell death, and may assist at designing therapies to expand the window for gene therapy intervention in LCA12 patients.

We first confirmed all the previously reported manifestations of the phenotype of the *rd3* mice^15^, with further attention to meaningful details. RetGC1 levels are drastically reduced in *rd3* retinas as reported, but can still be detected, and are estimated at ~12% of the wild-type levels in *rd3* mice at p20 (Fig. 1). RetGC1 is retained at proximal cell compartments, mostly at the inner segment and perinuclear regions (Figs. 1d and 4). GCAP1 and GCAP2 levels are reduced in *rd3* mice to ~37 and ~42% of wild-type levels. Although mostly retained at the inner segment as previously reported^15^, traces of RetGC1, GCAP1, and GCAP2 are also observed at the outer segment layer (Fig. 1d), explaining why *rd3* mice elicit bigger ERG responses than *rd3/rd3* GCAPs^−/−^ mice (Fig. 3).

The *rd3* strain of mice showed a fast rate of retinal degeneration, going from approximately ten rows of photoreceptor nuclei at p20 to approximately five rows at p44. GCAPs ablation substantially slowed down retinal degeneration, so that at 8 months of age *rd3/rd3* GCAPs^−/−^ mice still retained five rows of photoreceptor nuclei. Therefore, the loss of RD3 still caused cell death in the absence of GCAPs, but at a substantially slower rate. We infer that: (i) the loss of RD3 causes an initial insult, with its origin in the closure of CNG-channels and the ensuing chronic decrease in [Ca^2+^]_i_; and (ii) the GCAP proteins mediate/amplify the response to this initial insult.

At a molecular level we show that >75% of GCAP2 in *rd3* mice is phosphorylated and therefore in its Ca^2+^-free state, which is indicative of low [Ca^2+^]_i_ at *rd3* photoreceptor cell somas. Both GCAPs would therefore be in their Ca^2+^-free, guanylate cyclase activator state at photoreceptor cell somas of *rd3* mice, where they result in cellular damage. How would GCAPs induce damage in this context?

**Fig. 8:**
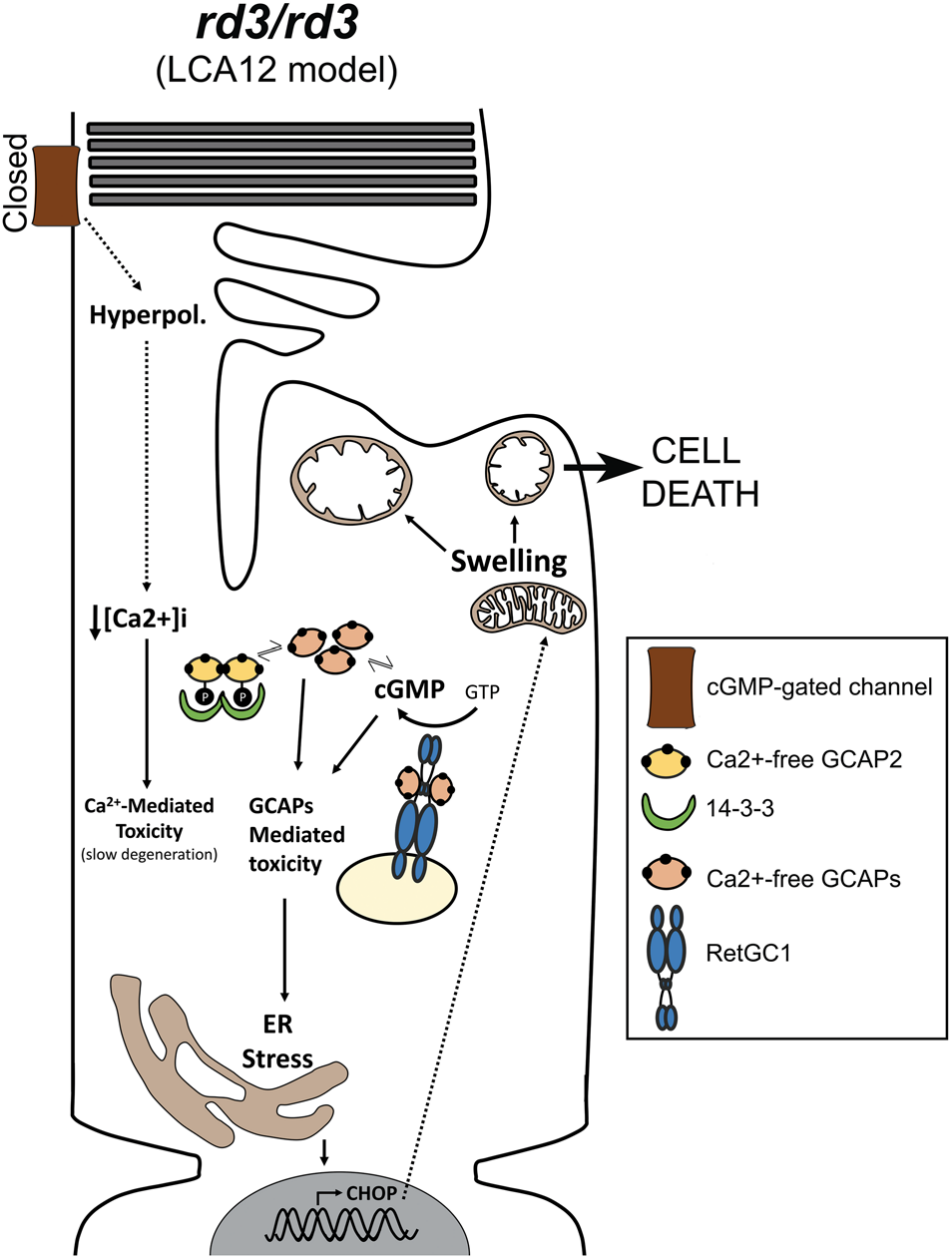
Sketch summarizing the mechanisms that likely contribute to the physiopathology of *rd3* mice and LCA12 patients. The lack of RD3 represents an immediate insult for photoreceptor cells by causing chronic hyperpolarization and chronic low [Ca^2+^]_i_ due to the closure of CNG-channels by the severe drop in cGMP synthesis. This chronic low [Ca^2+^]_i_ likely represents the original insult causing damage to the cell. Chronic low Ca^2+^ cause the GCAP proteins to remain permanently in their Ca^2+^-free conformation. GCAPs in their Ca^2+^-free guanylate cyclase activator state in the absence of RD3 may stimulate cGMP synthesis at the inner segment, ultimately causing ER stress and ER stress-mediated apoptosis. Ca^2+^-free GCAP2 conformational instability may also ultimately induce ER stress. GCAP2 phosphorylation and 14-3-3 binding might serve a protective role from both pathways of damage.

We envision two possible pathways, sketched in our proposed model in Fig. 8.

First, as proposed by Dizhoor et al.^29^ Ca^2+^-free GCAPs could, in the absence of RD3, stimulate RetGCs and promote constitutive cGMP synthesis at the inner segment, where it would result in toxicity. Dizhoor’s demonstration that RD3.GFP expressed as a transgene in rods localizes to the inner segment compartment^29^ supports RD3 proposed role of silencing RetGCs while at the inner segment^25^. Dizhoor et al. also bred the *rd3* mice to GCAPs^−/−^ mice and reported the retinal morphological rescue, interpreting the outcome as a demonstration of the importance of cyclase silencing at the inner segment for photoreceptor viability. While we believe that other scenarios cannot be discarded at this point, we consider our data to be consistent with Dizhoor’s interpretation. We here show that the levels of RetGC in *rd3* mice are ~12% of the wild-type levels, that are present at the inner segment layer. By showing that GCAP2 is mostly phosphorylated, we can infer that GCAPs are in their Ca^2+^-free form. Although 75% of the GCAP2 present in *rd3* mice (reduced to 42% of GCAP2 wild-type levels) is phosphorylated and bound to 14-3-3 and therefore not free to bind the cyclase, there is still ~10% of GCAP2 (referred to wild-type GCAP2 levels) that could bind the cyclase and activate it in a constitutive manner. Constitutive synthesis of cGMP would therefore rely on ~12% of the wild type levels of RetGC and ~10% of GCAP2, which is apparently low. However, constitutive synthesis of cGMP at the inner segment compartment of cones has been shown to be very toxic and the basis of the physiopathology in mouse models of achromatopsia caused by null mutations in cone CNG-channel subunits CNGA3 and CNGB3. In these models, activation of cGMP-dependent protein kinase (PKG) alters ER ionic homeostasis causing ER stress^32–34^. Actually, we here show that GCAPs mediate damage by activating ER stress-mediated apoptosis, by demonstrating CHOP induction as well as caspase 3 and PARP1 cleavage in *rd3* mice that are prevented in *rd3/rd3* GCAPs^−/−^ mice (Fig. 6). Nevertheless, evidence of cGMP synthesis specifically at the inner segment compartment and/or PKG involvement is still missing in the *rd3* model and will require further investigation. An intriguing thought, assuming that preventing RetGC activity at the inner segment is critical for photoreceptor’s viability, is that GCAP2 phosphorylation and 14-3-3 binding might serve as an additional mechanism to prevent cyclase activation in this compartment. Whether GCAP1 is phosphorylated is not known, and will require further investigation. However, it is GUCA1B gene encoding GCAP2 that was identified as a modifier gene of the *rd3* mouse phenotype^27^, and GCAP2 expression that was downregulated in *rd3* mice^35^.

A second putative pathway of damage involves the conformational instability of Ca^2+^-free GCAP2, manifested by its high tendency to aggregate when expressed in bacteria^26^. We have previously shown that a form of GCAP2 locked in its Ca^2+^-free form expressed as a transgene in rods is retained at the inner segment compartment by phosphorylation and 14-3-3 chaperone binding, leading to a fast retinal degeneration^26^. EF^−^GCAP2 toxicity occurs in the presence of RD3 silencing the cyclase. One possibility would be that the Ca^2+^-free form of GCAP2 formed toxic protein oligomers, with 14-3-3 binding serving a protective role by preventing this formation. In this manner, GCAP2 could cause damage in a similar way as how α-synuclein contributes to Parkinson’s disease^36,37^. The instability of Ca^2+^-free GCAP2 is also reflected in its high affinity for the HSP90 dimer observed in pull-down assays (Fig. 5), that raises the possibility that Ca^2+^-free GCAP2 under chronic low [Ca^2+^]_i_ may trigger the heat shock response.

A few other proteins were selectively identified as putative interactors of GCAP2-P, like the cytoskeletal regulator Ndel1, nuclear-distribution gene E homolog like-1 protein, a modulator of dynein that has been involved in securing the selectivity of cargo delivered to axons^38^, and at inhibiting primary cilia assembly in proliferating cells^39^. Ndel1 would be a putative candidate to mediate the morphological disc alterations observed in *rd3* mice. Future experiments will test this hypothesis, and aim at confirming other putative interactions.

Finally, by expressing RD3.V5 as a transgene in the rods of *rd3* mice, we report that RD3 localizes mainly to photoreceptor inner segments, although it also distributes to some extent to the outer segments (Fig. 4). The outer segment RD3 signal might not have been so apparent in the recent study of RD3.GFP localization in rods^29^ due to the protein modification by the much bigger tag. RD3 main localization to the inner segment provides some insight into the mechanisms underlying trafficking of the cyclase to the cilium. The cyclase is probably transported in two stages: a first stage, in which it is escorted and silenced by RD3 to the base of the connecting cilium; and a second, in which the GCAPs displace RD3 to bind the cyclase, previous to RetGC/GCAPs complex entry into the outer segment compartment. Future studies will be addressed at elucidating the mechanistic steps of this trafficking.

## Materials and methods

### Ethics statement

Pertaining to animal research, this study was conducted in accordance with the Association for Research in Vision and Ophthalmology (ARVO) statement for the use of animals in ophthalmic and vision research and in compliance with acts 5/1995 and 214/1997 for the welfare of experimental animals of the autonomous community (Generalitat) of Catalonia; ref. #9906, and approved by the ethics committee on animal experiments of the University of Barcelona, Bell 216/17, 217/17, and 218/17.

### Mice

The B6.Cg-Rd3rd3/Boc strain of *rd3* mice used in this study were obtained from the Jackson’s Laboratories (JAX Stock #8627 Maine, USA) as heterozygous wt/*rd3* mice^13^. These mice present a point mutation after residue 106 (C→T) that results in a premature stop codon after residue 106. The *rd3* mutation results in expression of a truncated form of the RD3 protein that is rapidly degraded^14^. Heterozygous animals were bred in order to obtain homozygous *rd3*/*rd3* mice. Mice were genotyped by real-time polymerase chain reaction (RT-PCR) by making use of the TaqMan® SNP genotyping assay (Life Technologies, Carlsbad, USA). For that purpose, forward and reverse primers were designed to amplify a fragment of 68 bp from the *Rd3* locus of genomic DNA that encompasses the *rd3* mutation (Fw: 5′-CTGGAGACGCTCATGATGGA-3′; and Rv: 5′-CGACGCTCCCTCTGTTGT-3′) and primer probes were designed to hybridize with either the amplified wild-type allele (VIC-5′-CTCTCTCATCTGCCCAGCC-3′) or knock-out allele (FAM-5′-CTCTCTCATCTACCCAGCC-3′). RT-PCR was performed in 25 μl reactions containing 12.5 μl TaqMan universal master Mix (Life Technologies); 300 nM each forward/reverse primer; 250 nM of each primer probe, and 20 ng of genomic DNA obtained from digested mouse tails (Nucleospin® Tissue, Macherey-Nagel, Düren, Germany). After 10 min incubation at 95 °C, thermal cycling was performed on the StepOne Real-time PCR System (Thermo Fisher Scientific, Waltham, Massachusetts) and consisted of 40 cycles (95 °C for 15 s; 60 °C for 1 min). End-point allelic discrimination genotyping was assessed by inspecting the fluorescence plots for the wild type versus mutated probe.

To obtain mice expressing the *rd3* mutation to homozygosis in the GCAPs^−/−^ background, *rd3*/*rd3* mice were bred to GCAPs^−/−^ mice. GCAPs^−/−^ mice lack GCAP1 and GCAP2 expression, and their phenotype has been described^40^.

### Antibodies

Anti-GCAP1 and anti-GCAP2 polyclonal antibodies have been previously described^22^. The polyclonal antibodies against RetGC1 and RD3 were generated in rabbit against C-terminal peptides corresponding to the last 21aa of murine RetGC1 and the last 16aa of murine RD3. Peptides were conjugated to keyhole limpet hemocyanin carrier protein (Imject maleimide activated carrier protein spin kit, Thermo Fisher Scientific) for inoculation in rabbits. A 90-day, three antigen-injection protocol was followed. Serum from immunized rabbits was purified by affinity chromatography by using the immunizing peptide covalently coupled to an agarose column by –SH chemistry (Sulfolink immobilization kit for peptides, Thermo Fisher Scientific). The specificity of the antibodies is shown by Western blot analysis of whole retinal extracts in Supplementary Fig. 1.

### Specimen preparation for light and electron microscopy

Mice of the indicated genotypes were reared under standard cyclic light (12 h dark:12 h light) and sacrificed by cervical dislocation. The vertical meridian of the eye was marked at the top for orientation purposes. The eye was enucleated, punctured, and immediately submerged in 2% paraformaldehyde, and 2.5% glutaraldehyde in 0.1 M cacodylate buffer pH 7.2 for 5 min. The cornea was excised and fixation was allowed to proceed for 1 h, before removal of the lense and further fixation of the eye cup for 12 h at 4 °C. Eye cups were washed with 0.1 M cacodylate buffer and fixed with 1% osmium tetroxide in 0.1 M cacodylate buffer for 2 h at room temperature. Specimens were dehydrated in ethanol (30–100%) or acetone, infiltrated with propylene oxide, and embedded in Epoxi embedding medium (Fluka Analytical, Munich, Germany). Retinal sections for histological analysis by light microscopy morphometry measurements were obtained with a Reichert Ultracut S ultramicrotome (Leica, Wetzlar, Germany) at 1-μm thickness and contrasted with Richardson’s stain (0.5% methylene blue, 0.5% Azine II, and 0.5% borax in dH_2_O). For transmission electron microscopy analysis, ultrathin sections were obtained (see below).

### Retinal morphometry analysis

For morphometrical analysis, measurements of ONL thickness were taken on 1-μm sections at the vertical meridian of the eye that encompassed the optic nerve. Overlapping frames at 20× magnification were acquired covering the whole section with a Zeiss Axio Zoom.V16 stereo microscope (Zeiss, Oberkochen, Germany). An integrated image of the whole retinal section was assembled with the HUGIN fusion software. Measurements of ONL thickness were taken at 200-µm intervals from the optic nerve (12 divisions in the superior retina, and 10 divisions in the inferior retina). For each morphometry analysis a minimum of four mice per genotype were used at indicated ages. Error bars represent the standard error of the mean (SEM).

### Ultrathin sectioning and image acquisition at the transmission electron microscope

Ultrathin sections (70–90 nm) were obtained with a Reichert Ultracut S ultramicrotome (Leica), collected on 200 mesh copper grids, counterstained by heavy metal staining (2% uranyl acetate in 50% ethanol for 30 min), and contrasted with 2% lead citrate for 10 min. Ultrathin sections were analyzed in a JEOL 1010 transmission electron microscope and images acquired with a Bioscan Gatan wide angle slow scan CCD camera.

### Immunofluorescence analysis in fixed tissue

For immunofluorescence analysis, eye cups were fixed in 4% paraformaldehyde, 0.02% glutaraldehyde in phosphate-buffered saline (PBS), pH = 7.4 for 2 h at room temperature. Eye cups were infiltrated in acrylamide (8.4% acrylamide, 0.014% bisacrylamide in PBS pH 7.4) overnight at 4 °C. Acrylamide polymerization was induced and eye cup acrylamide blocks immediately frozen in O.C.T. compound (Tissue-Tek, Electron microscopy Sciences, Hatfield, PA) using liquid nitrogen. Cryosections were obtained at 18-µm thickness with a CM15105 Leica Cryostat (Leica Microsystems, Wetzlar, Germany). Immunolocalization of GCAP1 and GCAP2 required an antigen retrieval protocol, consistent on incubation with proteinase K (0.05 mg/ml in PBS) for 2 min, followed by a 10 s heating shock at 70 °C. Sections were incubated with blocking solution (3% normal goat serum, 1% bovine serum albumin (BSA), 0.3% Triton-X100 in PBS pH7.4) for 1 h; first antibody in dilution buffer (3% normal goat serum, 0.4% BSA, 0.1% Triton-X100 in PBS pH 7.4) for 16 h at 4 °C; and secondary antibody (1.5 h at room temperature), and fixed with 4% paraformaldehyde prior to being mounted with Mowiol (Calbiochem #475904, San Diego, CA, USA). Antibodies used were anti-RetGC1 pAb; anti-GCAP1 pAb; anti-GCAP2 pAb; anti-rhodopsin mAb 1D4; anti-PNA-647 (# L32460, Thermo Fisher Scientific); anti-rabbit Alexa Fluor 488 (Thermo Fisher Scientific A-11034, Walthman, MA, USA); and anti-mouse Alexa Fluor 555 (Thermo Fisher Scientific #A-32727). Samples were mounted on 0.13–0.16 mm thick cover glasses.

### Confocal microscopy and data analysis

Confocal microscopy images were acquired at a confocal laser scanning microscope Zeiss LSM 880 equipped with one GaAsP detector, with a 63×/1.4 NA oil objective. For localization of RetGC and GCAPs in Figs. 1 and 2, spectral bands were set to 493–550 nm for the 488 channel, 566–638 nm for the 555 channel, and to 638–755 nm for the 647 channel. The *Z*-stacks covered 16 μm of tissue with a 368 nm step size (~40 planes/stack). The image acquisition settings provided a pixel size of about 0.132 × 0.132 μm in 1024 × 1024 images.

### Western blot

Retinas (at least *n* = 3 per condition) were homogenized in Laemmli sodium dodecyl sulfate (SDS) buffer with 1 mM phenylmethylsulfonyl fluoride (PMSF), and protease cocktail inhibitor (Complete mini EDTA-free, Roche). Samples were boiled for 10 min at 95 °C, and fractions corresponding to one tenth of a retina were resolved by 12 or 16% SDS–polyacrylamide gel electrophoresis. Proteins were transferred to nitrocellulose membranes (0.2 µm nitrocellulose, Bio-Rad, Hercules, CA, USA). Membranes were blocked for 1 h at room temperature with 5% non-fat dry milk in TBST and then were incubated overnight at 4 °C with custom polyclonal antibodies to RetGC1, RD3, GCAP1, and GCAP2, and antibodies anti-CHOP (Cell Signaling L63F7); anti-Caspase 3 (Cell Signaling 9662); anti-PARP1 (Cell Signaling 9542); goat anti-rabbit IgG (Heavy and Light chains) antibody Dylight™ 800 conjugated (Rockland #611-145-002, Pottstown, PA, USA); goat anti-mouse IgG (H&L) antibody Dylight™ 680 conjugated (Rockland #610-144-002-0.5); and donkey anti-goat conjugated to Horseradish Peroxidase (HRP) was from Thermo Fisher (86326). Detection was performed in an Odyssey Scanner (LI-COR Biosciences, Lincoln, Nebraska) or ImageQuant™ LAS500 (Ge Healthcare, Chicago, Illinois) image acquisition system. Band densitometric analysis was performed using the Fiji (Image J) software, and band intensity analysis presented in histograms always reflect band intensity of the protein of interest normalized by the intensity of tubulin in the loading control.

### Isoelectric focusing separation of murine retinal homogenates

Retinas from wt and *rd3/rd3* mice were dissected at p21, at an early onset of retinal degeneration of *rd3* mice. Retinas were homogenized in homogenization buffer with phosphatase inhibitors [20 mM Hepes, 115 mM KCl, 10 mM NaCl, 10 mM MgCl_2_, 50 mM NaF, 5 mM β-glycerophosphate, 1 mM PMSF, and a protease cocktail inhibitor (Complete mini EDTA-free, Roche, Basel, Switzerland); pH 7.4]. Samples were centrifuged at 13,200 rpm, 4 °C for 30 min, and supernatant fractions were kept. An aliquot was taken at this point for protein determination (BCA kit, Thermo Fisher Scientific). Protein material in supernatant fractions was precipitated by addition of three volumes of ice-chilled trichloroacetic acid (TCA)-acetone (13.3% w/v trichloroacetic acid in acetone, with dithiothreitol (DTT) to 20 mM final concentration); equilibration of samples to −20 °C for 72 h; and centrifugation at 13,000×*g* for 1 h at 4 °C. Protein pellets were washed twice with cold 20 mM DTT in acetone; and allowed to air-dry for 10 min. Samples were dissolved in urea/thiourea/CHAPS/DTT (7 M urea, 2 M thiourea, 40 mM DTT, 4% CHAPS, and 2% IPG buffer pH 3–10) to ensure complete reduction, denaturing, and solubilization of proteins in the samples.

Samples were kept at −80 °C until use. For isoelectric focusing (IEF) separation, gel strips with a preformed linear pH gradient of pH 3–10 (prehydrated IPG Immobiline (TM) DryStrips pH 3–10, 18 cm, GE Healthcare) were used. Threefold more sample was loaded for *rd3/rd3* retinas than wt retinas in order to equilibrate the GCAP2 signal. IEF separation was conducted on an Ettan IPGphor3 system (GE Healthcare), following a 500 V step for 1 h, a gradient to 1000 V for 1 h, a gradient to 10,000 V for 3 h, and a 10,000 V step for 3 h. Gel strips were incubated in transfer buffer for 15 min, and proteins were transferred to nitrocellulose membranes by capillary action. GCAP2 was immunoblotted with anti-GCAP2 pAb and goat anti-rabbit IgG (Heavy and Light chains) antibody Dylight™ 800. Bands were visualized at an Odyssey scanner (LI-COR), and densitometry analysis was performed with the Fiji (ImageJ) software.

### Expression, purification, and in vitro phosphorylation of myristoylated GCAP2 for pull-down assays

Myristoylated GCAP2 expression was induced in *Escherichia coli* BL21(DE3) cells transformed with pET-15b-bGCAP2 and the pBB131 plasmid encoding N-myristoyl transferase (a gift from Dr. J. Gordon, Washington University School of Medicine, Missouri, USA). Free myristic acid was added to 50 μg/ml to the cell culture and expression was induced for 4 h at 37 °C. bGCAP2 protein, was recovered from solubilized inclusion bodies and purified by on-column refolding using immobilized metal affinity chromatography (IMAC) on a Nickel-NTA [Nickel bound to agarose beads by chelation using nitrilotriacetic acid] as previously described^26^.

To obtain phosphorylated bGCAP2 for pull-down assays, in vitro phosphorylation reactions were performed with protein kinase G (PKGIα, Calbiochem, Billerica, MA, USA). Each 50 µl reaction contained 30 mM Tris-HCl pH 7.5, 5 mM MgCl_2_, 5 mM sodium phosphate buffer pH 7.5, 6 mM DTT, 2 mM EGTA, 10 µM ATP, and 500 µM cGMP, with 10 µg of bGCAP2 in the presence or absence of purified PKGIα (100 units) to obtain phosphorylated GCAP2 (GCAP2-P) and the mock-control (GCAP2). Phosphorylation reactions were allowed to proceed for 2 h at 30 °C, and GCAP2 and GCAP2-P were covalently linked to Epoxi-magnetic beads (Life Technologies, Carlsbad, CA, USA). GCAP2- or GCAP2-P-beads were then incubated with bovine retinal homogenates obtained by homogenization of fresh bovine retinas in binding buffer (10 mM HEPES, 135 mM NaCl, 5 mM KCl, 1 mM PMSF, 1 mM NaF, 1 mM β-mercaptoethanol, 1% Triton X-100, 4 mM EGTA, 2 mM EDTA, and Complete Mini protease inhibitors, pH 7.4). After 1 h incubation at room temperature, beads were washed and bound proteins were eluted under acidic conditions. Magnetic beads were extensively washed, and bound material was eluted in 0.2 M glycine pH 2.5 and immediately neutralized. Proteins in the bound fraction were identified by LC-MS/MS. The experiment included three biological replicates.

### LC-MS/MS

Samples were reduced with 10 mM DTT at 60 °C for 30 min, and alkylated with 55 mM iodoacetamide for 30 min at room temperature. Samples were precipitated with 10% TCA. Pellets were dissolved in 2 μl of 8 M urea and brought to a final volume of 10 μl with 25 mM ammonium bicarbonate. Samples were digested with sequencing grade trypsin in 25 mM ammonium bicarbonate for 12 h. For LC-MS/MS, samples were resuspended in 0.1% formic acid and injected into a series Proxeon LC nanoEASY system (Thermo Fisher Scientific) coupled to a LTQ-Velos Orbitrap (Thermo Fisher Scientific). The resulting mass spectral peak lists were searched with the Sequest search engine (v.2.1.04, Matrix Sciences, London, UK) against the merged BOVIN-MOUSE UP SP r 2011-1.fasta sequence library. For database searching, raw mass spectrometry files were submitted to the in-house MOUSE-BOVIN_UP_SP_r_2014-5.fasta Swiss-Prot database (released February 2014; 22460 protein entries) using SEQUEST version 28.0 (Thermo Fisher Scientific). The criteria used to accept identification included a minimum of two peptides matched per protein, with a false discovery rate of 1%. All proteins were treated as ungrouped.

### Characterizing differential protein interactions of GCAP2-P and GCAP2

For label-free quantitative proteomic analysis of proteins identified with GCAP2-P versus GCAP2, we first filtered the protein lists to remove any duplications resulting from the use of bovine and mouse fasta sequence libraries. Only those proteins unequivocally assigned by at least a unique peptide were retained. For each protein identified the NSAF was calculated with the following equation:

1 $${\mathrm{NSAF}}_k = \frac{{({\mathrm{SpC}}_k + 1)/L_k}}{{\mathop {\sum }\nolimits_{i = 0}^n ({\mathrm{SpC}}_i + 1)/L_i}}$$

where NSAF_*k*_ is the Normalized Spectral Abundance Factor^41^ for a given protein k; SpC_*k*_ is the Spectral Count for protein *k*, *L*_*k*_ is the length of the protein in number of amino acids; and the denominator is the summation of the NSAFs of all identified proteins in that sample. This expression corrects for differences in sampling depth between both conditions assayed, and avoids the discontinuity seen in simple count ratios when a protein shows spectral count = 0 in one of the samples.

Mean NSAF values were then calculated from triplicate biological samples, followed by a *t*-test comparison (two tails, unequal variance). To obtain the volcano plot, the ${\mathrm{log}}_2\frac{{{\mathrm{Mean}}_P}}{{{\mathrm{Mean}}_{NP}}}$ was plotted in the *x*-axis; and the $- 10{\mathrm{log}}(p\,value)$ from the *t*-test was plotted in the *y*-axis, with Mean_*P*_ referring to Mean NSAF_GCAP2-P_, and Mean_*NP*_ referring to Mean NSAF_GCAP2._ Threshold curves for the volcano plot were obtained by fitting values to

2 $$f\left( x \right) = \frac{1}{{x^2 - n}} + m$$

were *n* is the value of the ${\mathrm{log}}_2\frac{{{\mathrm{Mean}}_P}}{{{\mathrm{Mean}}_{NP}}}$ ratio threshold and *m* is the −10log(*p* value) threshold. *m* and *n* values were fixed to 5 and 1.35 respectively. (Notes: taking $a \equiv {\mathrm{log}}_2\left( {\frac{P}{{NP}}} \right)$ and $b \equiv - 10{\mathrm{log}}(p - value)$, the proteins passing the thresholds are those proteins that: $a < - \sqrt n$ and *b* > *f*(*x*) for the negative side; and $a > \sqrt n$ and *b* > *f*(*x*), for the positive side.

### Electroretinography

A total of 4–7 mice were tested for each animal group. Dark-adapted (>12 h) animals were anesthetized with an intraperitoneal injection of ketamine (70 mg/kg; Ketalar, Parke-Davis, Wellington, New Zealand) and xylazine (7 mg/kg; Rompun, Bayer, Leverkusen, Germany) in saline solution (NaCl 0.9%) and pupils were dilated with one drop of 1% tropicamide. A corneal electrode was used to record ERGs from right eyes (Burian-Allen, Hansen Ophthalmic Development Lab, Coralville, IA). Electrode was placed in the visual axis 1–2 mm from the cornea and a drop of 2% methyl-cellulose (Methocel, Ciba Vision, Hetlingen, Switzerland) was dropped between cornea and electrode. Mice were maintained for >5 min in absolute darkness before the recordings. Mouse temperature during the recording was maintained at 37 °C with a liquid heating pad. Full-field flash ERG responses were recorded with the retina illuminated with a LED-driven Ganzfeld dome. A series of light flashes of increasing intensity (from 0.001 to 10 cd/s/m^2^) were averaged both in scotopic and photopic conditions. Photopic cone responses were recorded following 5 min of light adaptation with a background white light (50 cd/m^2^). Light intensity was controlled for each animal group (Mavo-Monitor USB, Gossen, Germany). Recorded electrophysiological responses were amplified, filtered (CP511 AC amplifier; Grass Instruments, Quincy, MA), and digitalized (ADInstruments Ltd, Oxfordshire, UK). The recording process was controlled with Scope version 3.8.1 software (Power Lab, ADInstruments Ltd). The stimulation protocols were designed according to the International Society for Clinical Electrophysiology of Vision.

### Generation of pRho-mRd3.V5-dsRed expression vector and in vivo DNA electroporation

The expression vector used for in vivo DNA electroporation was based on the plasmid pRho-DsRed. pRho-DsRed was a gift from Connie Cepko (Addgene plasmid#11156; http://n2t.net/addgene:11156;RRID:Addgene_11156)^42^. This plasmid contains the 2.2 kb-version of the rod opsin promoter from Bos taurus^43^; a multicloning site; a Kozak sequence; the DsRed2 reporter gene; and the β-globin poly(A) signal^42^. To make pRho-mRD3.V5-DsRed, murine *rd3* cDNA was amplified by PCR with a forward primer that introduced an XbaI restriction site and the reverse primer introducing the V5 epitope in frame with RD3 COOH-terminal sequence, followed by an EcoRI restriction site. The mRD3.V5 DNA was inserted in the XbaI and EcoRI sites of pRho-DsRed, and the resulting expression vector was verified by sequencing. For in vivo electroporation, pRho-mRd3.V5-dsRed vector was amplified with the pureLinkTM Expi Endotoxin-Free Mega Plasmid Purification Kit (Invitrogen, Carlsbad, California).

In vivo DNA electroporation in the retina following DNA injection in the subretinal space was performed as originally described^42^ with minor modifications. A DNA solution (6 μg/μl) was prepared by mixing pRho-DsRed and pRho-mRD3.V5-DsRed at a molar ratio of 1:4 in phosphate saline buffer with 0.1% v-v fast green. Even though pRho-mRD3.V5-DsRed expressed dsRed, we found the levels of expression too low for detection of the eye injected area at the fluorescence dissection scope. Therefore pRho-DsRed was coinjected with pRho-mRD3.V5-DsRed. Pups were injected the day they were born at the subretinal space, with 0.2–0.5 µl of DNA by using a Hamilton syringe with a blunt 30-gauge needle under a dissecting microscope (Zeiss KL1500LCD. Stemi2000, Germany). Tweezer-type electrodes were placed softly holding the head of the pup, and five 80 V square pulses of 50 ms duration with 950 ms intervals were applied using a CUY21 electroporator (Nepagene, Chiba, Japan). Electroporated eyes were processed at p20–p25 for immunofluorescence localization analysis as described above. Antibodies were anti-RetGC1 pAb and the anti-V5 mAb (2F11F7, Invitrogen) by secondary antibodies α-rabbit Alexa Fluor 488 (Life Technologies) and α-mouse Alexa Fluor 647 (Life Technologies) both at 1:500 for 1.5 h at room temperature. The image has been acquired with a LEICA SP5 microscope using a 63 × 1.4 NA oil objective. The pixel and step sizes are 100 nm and 198 nm respectively providing an effective image volume of 131 μm × 131 μm × 9.5 μm. Two Hybrid detectors in photon counting mode and a photomultiplier tube (PMT) have been used to acquire the different channels. The spectral configuration for the channel 1 used a 488 nm laser line and an emission window from 498 to 535 nm. The channel 2 used the 633 nm laser line and an emission window between 642 and 722 nm. Finally, the channel 3 used the 543 nm laser line with the emission window set between 551 and 616 nm.

## Supplementary information

SUPPLEMENTARY MATERIAL:

Supplementary Table 1
