# Supplementary material for: GCAP neuronal calcium sensor proteins mediate photoreceptor cell death in the rd3 mouse model of LCA12 congenital blindness by involving endoplasmic reticulum stress
Source: Cell Death Dis. 2020 Jan 24;11(1):62. doi: 10.1038/s41419-020-2255-0 (PMC6981271; doi:10.1038/s41419-020-2255-0)
Supplement: Supplementary file 1 — SUPPLEMENTARY MATERIAL: [file 41419_2020_2255_MOESM1_ESM.docx]

**SUPPLEMENTARY MATERIAL:**

**Fig S1.** Specificity of the polyclonal antibodies generated in rabbit against murine RetGC1 and murine RD3. Whole retinal homogenates from murine retinas (material corresponding to one tenth of a retina) were resolved by 12% SDS-PAGE, transferred to nitrocellulose and immunoblotted with affinity purified anti-RetGC1 or anti-RD3 and a goat anti-rabbit IgG (H&L) antibody Dylight™ 800 conjugated. Detection was performed in an Odyssey Scanner (LI-COR) image acquisition system.


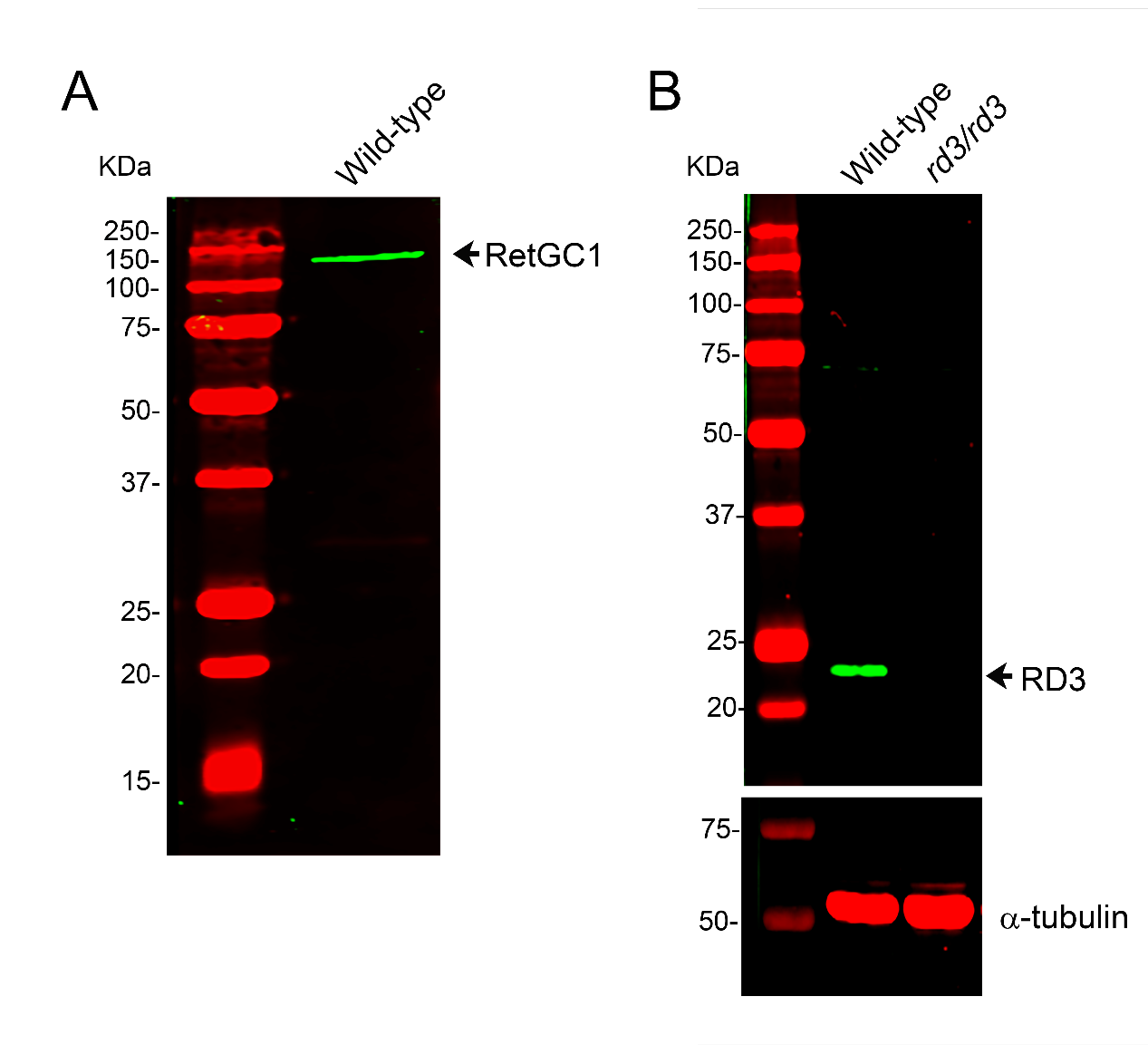


Fig S1

**Fig S2.** Uncropped blots with molecular weight marker lanes for the Western blots presented in Fig 1 and Fig 2.


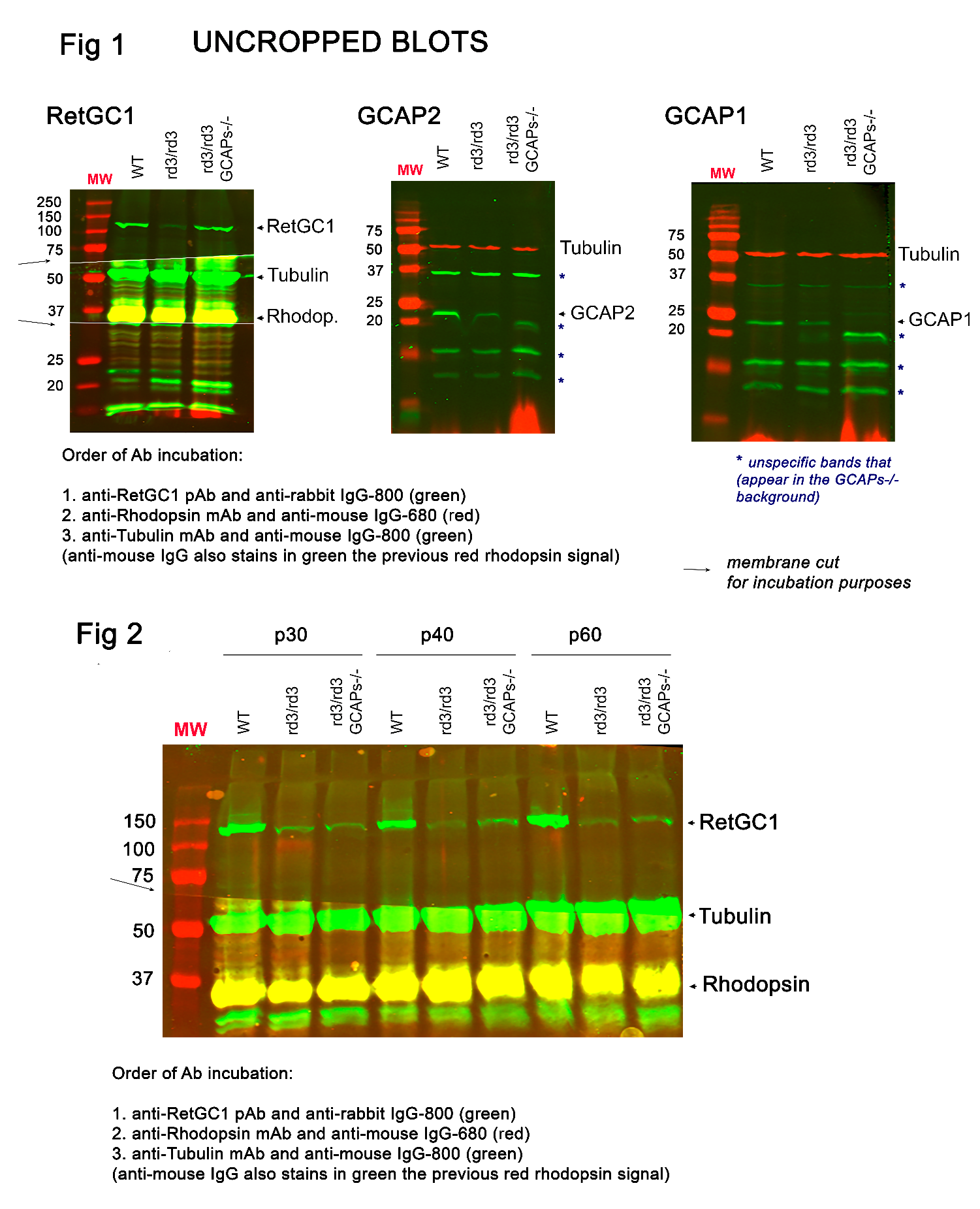


**Fig S3.** Uncropped blots with molecular weight marker lanes for the Western blots presented in Fig 6.


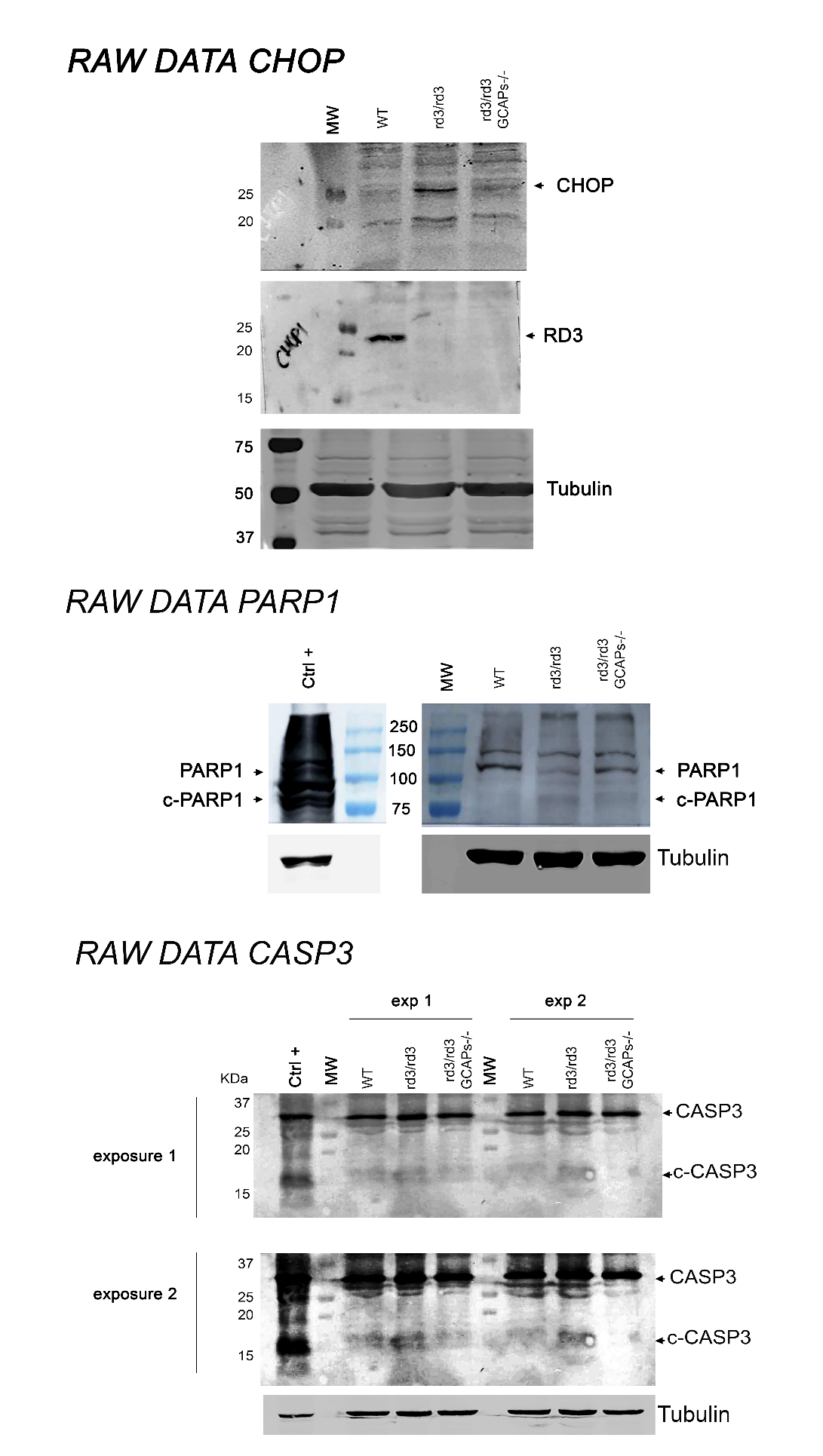


**Supplementary Table 1.** List of proteins identified by LC-MS/MS in pull-down assays with purified recombinant myristoylated Ca^2+^-free GCAP2 (NP, non-phosphorylated) or GCAP2-P (phosphorylated) on bovine retinal extracts, indicating the spectral count and number of total and unique peptides identified for each protein. Pull-down assays were performed in triplicate. The dataset shows the calculation of the normalized spectral abundance factor (NSAF) for each protein in each sample, and application of the statistical analysis detailed in Methods that results in the Volcano plot presented in Fig 5C.
